# Major Bioactive Compounds, Volatile and Sensory Profiles of *Coffea canephora* Flowers and Infusions for Waste Management in Coffee Production

**DOI:** 10.3390/foods14060911

**Published:** 2025-03-07

**Authors:** Juliana DePaula, Sara C. Cunha, Fábio Luiz Partelli, José O. Fernandes, Adriana Farah

**Affiliations:** 1 Laboratório de Química e Bioatividade de Alimentos & Núcleo de Pesquisa em Café Professor Luiz Carlos Trugo-NuPeCafé, Instituto de Nutrição Josué de Castro, Universidade Federal do Rio de Janeiro, Rio de Janeiro 21941-902, Brazil; 2 LAQV/REQUIMTE, Laboratório de Bromatologia e Hidrologia, Departamento de Ciências Químicas, Faculdade de Farmácia da Universidade do Porto, 4099-030 Porto, Portugal; sara.cunha@ff.up.pt (S.C.C.); josefer@ff.up.pt (J.O.F.); 3 Departamento de Ciências Agrárias e Biológicas, Centro Universitário do Norte do Espírito Santo, Universidade Federal do Espírito Santo, São Mateus 29932-900, Espírito Santo, Brazil; partelli@yahoo.com.br

**Keywords:** conilon, robusta, novel food, chlorogenic acids, caffeine, volatile organic compounds, aroma, flavor

## Abstract

This study aimed to investigate the content of major bioactive compounds and characterize the volatile and sensory profiles of *Coffea canephora* flowers and their infusions. Dried flowers from six selected genotypes of *C. canephora* trees and their infusions were analyzed for bioactive compounds using HPLC–DAD, while volatile organic compounds (VOC) were analyzed using GC–MS. Eight chlorogenic acids (CGA), seven phenolic acids, and the alkaloids caffeine and trigonelline were quantified in all methanolic flower extracts. Total CGA, phenolic acids, caffeine, and trigonelline contents in the methanolic extracts ranged between 342.8 and 1079.4 mg/100 g, 27.1 and 41.0 mg/100 g, 515.6 and 745.9 mg/100 g, and 453.8 and 645.2 mg/100 g, respectively. CGA, caffeine, and trigonelline were well extracted (84%, 91%, and 74%, respectively) when the flowers were infused in hot water. No free phenolic acids were identified in the infusions. Eighty-five VOC were identified in the flowers. Aldehydes, monoterpenes, esters, alcohols, monoterpene alcohols, acids, and ketones prevailed in order of the number of compounds. In the infusions, 38 VOC were accurately identified. Monoterpenes and monoterpene alcohols prevailed. In general, floral, jasmine and orange blossom, herbal, green coffee, woody, and sweet were the most cited sensory attributes for fragrance, aroma, and flavor. Considering the typically weak aroma of *C. canephora* seeds, the aroma and flavor of the flower’s infusions were surprisingly strong and pleasant, showing great marketing potential.

## 1. Introduction

*Coffea canephora* is one of the main species commercially explored worldwide, accounting for about 40–45% of the coffee market [1]. Vietnam is the world’s largest producer and exporter of *C. canephora* cv. robusta. Brazil is the second largest producer of this species, mainly cultivating the conilon cultivar [2]. Together, Vietnam and Brazil account for about 69% of world *C. canephora* production, while Indonesia, Uganda, and India account for about 12%, 6%, and 6%, respectively. The remaining percentage is spread among smaller producers [1,2]. This coffee species is more tolerant to heat and more resistant to climate change, being recently valued in the context of global warming.

**Figure 1:**
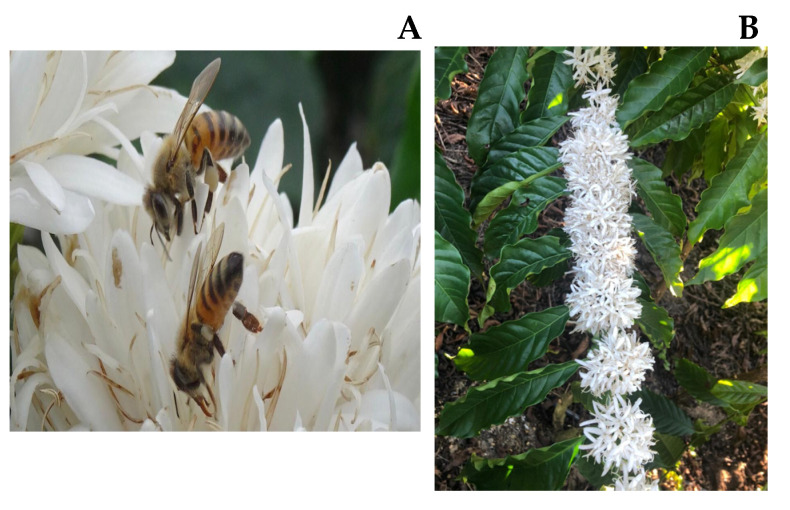
(**A**) Pollination of *Coffea canephora* flowers. (**B**) Flowers ready for harvest.

*C. canephora* shrubs are perennial and are pruned to reach a maximum height of 3 m to facilitate the fruit harvest. The flowers are rapidly pollinated and fall about 48 h after blooming [3]. Following, the ovaries slowly develop into “pinhead fruits”, a small infructescence that gives rise to fruits (Figure 1). The coffee tree flower is crucial for the quantity and quality of the fruits because it makes pollination possible, and, consequently, production [4]. Despite many beliefs, harvesting the flowers does not result in low productivity of seeds when they are plucked after pollination when the flowers start becoming light brown and wilting off the plant before the formation of the “pinhead fruits” [5,6].

The coffee flower can be divided into three main components: pollen, nectar, and petals. The pollen is rich in proteins, amino acids, carbohydrates, and saturated and unsaturated fats. The nectar mostly contains simple sugars (glucose, fructose, and sucrose), amino acids, proteins, inorganic ions, alkaloids, lipids, and organic acids. The petals and other parts contain vitamins, minerals, and antioxidants, especially polyphenols and other compounds that are potentially beneficial to health, such as caffeine and trigonelline [6,7,8,9].

Tons of coffee flowers fall to the ground annually and are wasted because of a lack of workforce to collect them or because their health and sensory potential are unknown. The Food and Agriculture (FAO) adopted the Sustainable Development Agenda for 2030 as a plan of action that provides a vision for a world that includes developing more efficient, inclusive, resilient, and sustainable agrifood systems [10]. In this sense, agrifood waste can be a relevant source of nutrients and bioactive compounds, providing multiple health benefits and adding value to the production chain [11]. Linked to this, there is a growing interest in the consumption of unconventional plants, like flowers, in human´s fed [12,13,14].

In recent years, several studies have revealed the nutritional, medicinal, and sensory relevance of flowers that are commonly overlooked, especially in conventional Western cultures. The coffee flower’s aroma, flavor, and bioactive potential make it appealing for use as an infusion. Lately, *Coffea arabica* flowers have been marketed in the West as “coffee blossom tea” after drying, by a few producers, but little is known about the consumption potential of *C. canephora*, especially because this species has been traditionally undervalued due to the “inferior” sensory quality of the seeds compared to *C. arabica*. Herbal and stimulant teas are among the most popular beverages in the world, with a long history of use as medicinal and functional beverages [15]. In 2023, the global consumption of tea amounted to about 7.3 billion kilograms and is estimated to reach 8.3 billion kilograms by 2029 [16]. Brazil follows the global trend, with a 54% increase in daily *per capita* consumption in the last decade [17]. The increased awareness and concern for health, as well as positive changes in the sensory attributes of appearance, taste, and aroma, and even in packaging, may explain this trend in Brazil and globally [18].

Marketing coffee flowers would potentially benefit consumers and producers, aggregating value to coffee production. Nevertheless, although different parts of the *Coffea* spp. plant are or have been traditionally consumed in several producing countries (including the flowers in the Yemen region) [19], the coffee flower is most likely to be classified in the EU as a “Novel Food”, meaning that it has not been consumed significantly in the European Union before 1997 and, therefore, it requires marketing authorization [20]. For this, scientific information involving the determination of chemical composition, microbiological and toxin screening, and safety assessment proving that people who had previously consumed the product did not develop health problems is required [21]. The conversion of coffee by-products into health promotion products is an exciting possibility for coffee farmers and consumers worldwide as a way of supporting sustainability in coffee production, providing that the product follows safety rules defined by regulatory agencies [22].

Research on chemical characterization in the field of coffee flowers is still lacking, especially from *C. canephora* plants. In 2019, Nguyen et al. [8] analyzed a few bioactive compounds in coffee flowers collected from robusta coffee farms in Vietnam. In 2021, de Abreu Pinheiro et al. [6] identified caffeine, trigonelline, gallic acid, and 5-caffeoylquinic acid in flowers from *C. arabica* and *C. canephora* cv. conilon plants harvested in Brazil. In 2022, Wirz et al. [9] quantified organic acids, trigonelline, caffeine, 5-caffeoylquinic acid, and 3,4 and 3,5-dicaffeoylquinic acids in 35 samples of *C. arabica*, *C. canephora*, and *C. liberica* flowers from El Salvador, Malaysia, India, and Thailand. No data have been found on the characterization of all major chlorogenic acid compounds present in coffee (caffeoylquinics, feruloylquinics, and dicaffeoylquinics) in *C. canephora* cv. conilon genotypes.

Furthermore, despite the highly recognizable scent, to date, there is only one related dataset reporting on the volatile profiles of *C. arabica* flowers at different annual rainfalls [23], one report on the volatile profile of *C. canephora* cv. robusta flowers [24], and one sensory testing of coffee flower infusions from *C. arabica*, *C. canephora*, and *C. liberica* flowers from different countries [9]. No data have been found on the characterization of the conilon cultivar and the relationship between the chemical and sensory characterization in coffee flower infusions.

Considering all of the above, this present study investigated the content of the major bioactive compounds and characterized the volatile and sensory profiles of *C. canephora* cv. conilon flowers and their infusions as part of an effort to value and consolidate their consumption worldwide.

## 2. Materials and Methods

### 2.1. Samples

**Figure 2:**
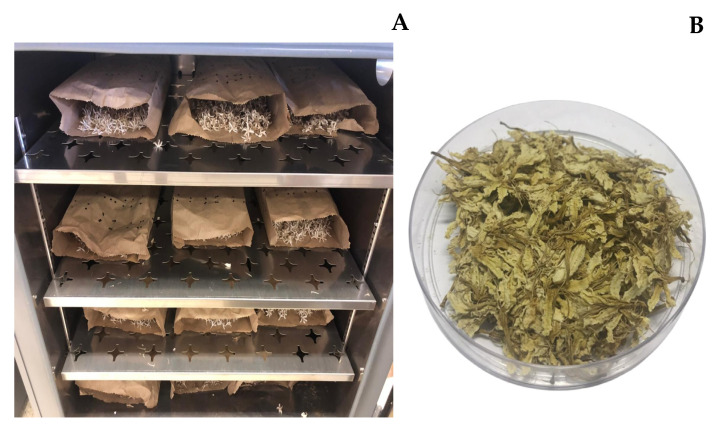
(**A**) Drying of *Coffea canephora* flowers. (**B**) Dried flowers.

Composites of genotypes of *C. canephora* trees (Verdim R, B01, Bicudo, Alecrim, 700, CH1) were grown in Nova Venécia, Espírito Santo, Brazil, at latitude 18°39′43″ south and longitude 40°25′52″ west, 199 m of altitude and an annual average temperature of 23 °C. The region has a tropical climate, characterized by a hot and humid summer and dry winter, classified as Aw, according to the Köppen classification [25]. The coffee flowers were harvested and dried in a forced circulation oven at 35–40 °C for 3 days in paper bags (Figure 2).

### 2.2. Water Content

In order to express the contents of nonvolatile compounds on a dry weight basis (db), the water content of the dried flowers (expressed as percentage) was determined using an MX-50 moisture analyzer (A&D Company, Limited, Tokyo, Japan).

### 2.3. Infusion Preparation

Infusions (n = 6) were prepared as described in DePaula et al. [22,26].

### 2.4. Physicochemical Analyses

Instrumental color of the infusions, soluble solids, pH, and titratable acidity were determined as described in DePaula et al. [22,26].

### 2.5. Analyses of Bioactive Compounds

Extractions and analyses of bioactive compounds in flowers and infusions were performed as described in Farah et al. [27]. Identification was performed with a liquid chromatographer coupled to a mass spectrometer (LC–MS), UV spectra, and standards. The quantification was performed with HPLC–DAD and external standard curves, as thoroughly explained in Farah et al. [27].

### 2.6. Analysis of Volatile Organic Compounds (VOC)

The extraction of VOC from flowers and infusions was performed by headspace solid-phase microextraction (HS-SPME). Qualitative analyses were performed using gas chromatography as described by Wang et al. [28], with adaptation reported in DePaula et al. [26].

### 2.7. Sensory Characterization

Infusions were characterized by a sensory panel consisting of nine trained assessors (aged 28–58) from Brazil and the USA, with a minimum of 200 h of experience in evaluating different food products and 50 h of experience in evaluating teas or infusions. In order to generate sensory descriptors, six samples of *C. canephora* flower infusions were presented to the assessors. The infusions were prepared as described in Section 2.3, served at 68 ± 2 °C [29,30,31], and evaluated. The trained panel generated their individual descriptors using a modified grid method [32]. Via open discussion, the panel leader agreed on the best descriptors to fully describe the samples, their definitions, and how to evaluate those [33].

### 2.8. Statistical Analysis

Data from physical and chemical analyses were processed using Statistica^®^ (Version 13.4.0.14) and presented as mean ± standard deviation. They were compared for differences with one-way ANOVA, followed by the Fisher test, at a 5% significance level. The Pearson correlation was used to correlate bioactive compounds in the flowers and soluble solids, color parameters, and bioactive compounds in the infusions.

## 3. Results and Discussion

### 3.1. Water Content and Bioactive Compounds in Coffee Flowers

Table 1 contains data from water content and main bioactive compounds in the coffee flower methanolic extracts evaluated in this study. On average, after drying, the water content in the flowers ranged from 6.1 to 7.6%. This percentage was used to express the content of the bioactive compounds on a dry basis (db).

Eight chlorogenic acid compounds (CGA), seven phenolic acids, and the alkaloids caffeine and trigonelline were quantified in all flower extracts (Table 1). CGA are the main phenolic compounds present in the seeds and other parts of the coffee plant, such as husks and leaves (around 90%) [34,35]. The presence of CGA, caffeine, and trigonelline is expected in all parts of the *Coffea* species. These compounds are known to influence the flavor of the coffee seed and are directly related to its antioxidant and anti-inflammatory actions in vivo and, consequently, to its beneficial health properties [34,35].

Eight CGA compounds were quantified in all *C. canephora* flower extracts evaluated: 3-CQA, 4-CQA, 5-CQA, 4-FQA, 5-FQA, 3,4-diCQA, 3,5-diCQA, and 4,5-diCQA. Total CGA contents in all flower extracts ranged between 342.8 and 1079.4 mg/100 g db (Table 1). The data available for comparison investigated only 5-CQA, which was not identified by de Abreu Pinheiro et al. [6] when evaluating a sample of freeze-dried and dehydrated conilon coffee flower (from Conceição do Castelo, Espirito Santo, Brazil),but was identified and quantified in a sample of freeze-dried arabica coffee flower (from Venda Nova do Imigrante, Espírito Santo, Brazil) (74.3 ± 3.4 mg/100 g db) and in one sample of dehydrated arabica coffee flowers (7.0 ± 0.5 mg/100 g db). 5-CQA has been identified and quantified in one sample of *C. canephora* cv. old paradenia from India (80 mg/100 gdb) [9]. These amounts are lower than those observed for 5-CQA in this study (224.8–708.0 mg/100 g db, Table 1). Nguyen et al. [8] did not identify this compound in any of the two robusta coffee flower samples (from Tay Nguyen, Vietnam) evaluated. Wirz et al. [9] also reported higher contents of 3,4-diCQA (11–252 mg/100 g) and 3,5-diCQA (11–252 mg/100 g) than those observed in this study (7.9–24.8 mg/100 g db and 25.0 and 78.8 mg/100 g db, respectively, Table 1).

Regarding the distribution of CGA classes and isomers in the flowers, as in the seeds, CQA isomers were the most abundant compounds, representing about 80% of total CGA, with 5-CQA being the main isomer (66% of total CQA). DiCQA isomers accounted for about 13% of the total CGA, with 3,5-diCQA being the major isomer (55% of total diCQA), followed by 4,5-diCQA and 3,4-diCQA. Total FQA represented about 7% of CGA, with 5-FQA corresponding to approximately 90% of FQA isomers; 3-FQA was not identified in this study. The predominance of 5-CQA and 3,5-diCQA in the seeds and leaves of the genus Coffea has been consistently reported in the literature [34,35,36,37,38], as well as the absence of the 3-FQA isomer in the leaves [36,37,38]. Considering that the exposure of CGA to high temperatures during drying or toasting/roasting tends to lead to the formation of CGA lactones, as previously observed in roasted coffee seeds [39] and in toasted maté leaves [40], the presence of CGA ɣ-lactones was investigated. However, no amount was identified in the dried flower extract since drying was performed at a maximum temperature of 40 °C.

Regarding phenolic acids, caffeic acid, ferulic acid, *p*-coumaric acid, vanillic acid, gallic acid, and 3,4-dihydroxy benzoic acid were detected in all *C. canephora* flower extracts (Table 1). Total phenolic acid contents ranged between 25.2 and 41.0 mg/100 g db (Table 1). Nguyen et al. [8] have previously identified in dehydrated robusta coffee flowers higher contents of gallic acid and 3,4-dihydroxybenzoic acid (mean of 77.5 and 28.7 mg/100g db, respectively) than those observed in this study. Similar gallic acid contents to those observed in this study were also identified in dehydrated conilon coffee flowers by de Abreu Pinheiro et al. [6] (mean of 3.9 mg/100 g db). Rutin, quercetin, and kaempferol (limit of quantification 2–3 µg/100 g) were investigated and not identified in the extracts.

Caffeine contents ranged between 515.6 and 745.9 mg/100 g db (Table 1). These contents are similar to those reported by Wirz et al. [9] for dehydrated *C. canephora* cv. old paradenia (from India) (500.1 ± 10.0 mg/100gdb) and lower than those reported by Nguyen et al. [8] for dehydrated robusta coffee flowers (1070.8 ± 0.4 mg/100 g db) and by de Abreu Pinheiro et al. [6] for dehydrated conilon coffee flowers (2754.9 ± 0.4 mg/100 g db).

Trigonelline contents ranged between 453.8 and 645.2 mg/100 g db (Table 1). These contents are lower than those reported by Wirz et al. [9] for dehydrated *C. canephora* cv. old paradenia (from India) (1500 ± 0.0 mg/100 g db), by Nguyen et al. [8] for dehydrated robusta coffee flowers (1092.8 ± 0.1 mg/100 g db), and by de Abreu Pinheiro et al. [6] for dehydrated conilon flowers (6258. 3 ± 351.9 mg/100 g db).

No correlation was found in the content of all the bioactive compounds.

It is worth noting the differences in the chemical composition of different genotypes, even though they were grown in the same edaphoclimatic conditions. *C. canephora* plants are characterized by having diploid cells (2n = 2) and 22 chromosomes, and their reproduction occurs through allogamy or cross-pollination, with the participation of two gametes [41,42]. The reproductive self-incompatibility of the *C. canefora* plants and the consequent inability of self-fertilization or pollination between plants with similar reproductive gamete organization leads to greater genetic variability, greater diversity of characteristics [3,41], and lack of standardization in the chemical compositions of these genotypes.

### 3.2. Physicochemical Analyses of Infusions

Table 2 contains the visual appearance, instrumental color, soluble solids, pH, and titratable acidity of the coffee flower infusions evaluated in this study. The L* values of infusions varied from 85.90 to 89.44. a* values were negative in all samples, varying between −4.18 and −4.32. b* values were positive in all samples, varying between 8.22 and 8.45. These results indicate that *C. canephora* flower infusions were light, greenish, and had shades of yellow, as visually and numerically perceived in Table 2.

In this study, the soluble solid values (0.2 °Brix, using 1 g flower/100 mL) were similar in all coffee flower infusions. pH ranged from 5.3 to 5.6, and TA values from 0.08 to 0.10 mEq NaOH/L. These results are similar to those previously reported for coffee leaf tea (0.2 °Brix) (using 1 g leaf/100 mL) [22] and showed the intermediate acid characteristics of *C. canephora* flower infusions.

### 3.3. Major Bioactive Compounds in Coffee Flower Infusions

Table 3 contains the major bioactive compound data of the coffee flower infusions evaluated in this study.

All eight CGA compounds identified in the methanolic extracts were also identified in the infusions. Total CGA contents in infusions ranged between 2.8 and 9.2 mg/100mL. Considering the methanolic extract results, this amount represents about 84% of extraction during the infusion preparation. This is equivalent to about 8% of the total CGA content reported by Cerca et al. [44] for *C. canephora* brews prepared by manual hot dripping at a concentration of 5%. No free phenolic acid was identified in the infusions.

Caffeine and trigonelline contents in infusions ranged between 4.7 and 7.1 mg/100 mL and between 3.5 and 4.8 mg/100 mL, respectively. On average, about 91% of caffeine and 74% of trigonelline were extracted when the flowers were infused in hot water. This is equivalent to about 7% and 12%, respectively, of caffeine and trigonelline contents reported by Cerca et al. [44] for *C. canephora* brews prepared by manual hot dripping at a concentration of 5%.

No correlation was found among bioactive compounds, and between each of them and their their color parameters, or their soluble solid content.

### 3.4. Volatile Organic Compounds (VOC) in Coffee Flowers and Infusions

Table 4 presents the volatile compounds identified in coffee flowers and their infusions, as well as their classical odor description.

#### 3.4.1. VOC in Coffee Flowers

Considering all *C. canephora* flower samples, 85 VOCs (corresponding to 94–98% of the total peak areas of the chromatograms) were accurately identified (Table 4). They were grouped into 10 chemical classes: 23 aldehydes, 15 monoterpenes, 13 esters, 13 alcohols, 7monoterpenes alcohols, 5acids, 4ketones, 3furans, 1pyrazin, and 1 organosulfur compound. Of the 85 compounds, 39 compounds were common to all flowers, including 20 potential impact ones (meaning whose odor can be perceived at very low concentrations), according to reports in the literature obtained by GC-olfactometry and/or their Odor Activity Values (OAV) (Table 4). The remaining compounds were distributed among the different genotypes.

From the 85 compounds identified in the flowers, 25 were also reported for coffee husks [26] and 35 for dried leaves [22]. Mostly aldehydes, alcohols, and monoterpene alcohols were among the common compounds previously found in the different parts of the coffee plant. In this work, most of the compounds identified in the flowers (and not previously in husks and leaves) were identified in all genotypes. However, some compounds were unique to specific genotypes, which probably contributed to differences in the sensory results among the genotypes. This will be explored later. Despite the large variation in the volatile profiles among samples, most identified compounds have been reported by Hafsah et al. [24], who examined flowers from *C. canephora* cv. robusta grown in Indonesia. A few compounds reported by these authors have been detected in this study but did not meet the applied peak confirmation criteria; therefore, they were not considered.

Although the area does not directly reflect the concentration of the compound, it is a good indication of its quantitative importance [22].

Considering the six *C. canephora* flowers, aldehydes represented about 14.1–26.3% of the total peak area of chromatograms. Aldehydes contribute remarkably to citrus, fruity, floral, fresh, and herbaceous notes (Table 4). Of 23 aldehydes, 14 were common to all samples. Some of these compounds, such as heptanal, octanal, nonanal, and citronelal, have been listed as impact compounds in citrus fruits and have attractive sensory qualities, according to aroma and flavor assessments [56]. Benzaldehyde and hexanal were also identified in all genotypes (Table 4). They are potential impact compounds synthesized in plants via the benzoic acid β-oxidative [57] and lipoxygenase pathways [58], respectively, and are listed as key aroma and flavor compounds in black tea [28,50,51,52]. Two additional impact compounds were identified: decanal in genotypes Verdim, Alecrim, 700, and CH1 and dodecanal in genotype 700.

Monoterpenes comprised 4.7–9.1% of the total VOC peak areas. Although monoterpene compounds have a poor aroma, they still impart sweet, citrus, fruity, woody, and herbal characteristics [59] (Table 4). Of 15 monoterpenes identified, 5 were common to all samples: β-myrcene, β-ocimene, α-phellandrene, *trans*-alloocimene, and α-terpinene. High concentrations of β-myrcene and β-ocimene were observed in a study on volatile compounds of different citruses’ flowers [60]. β-myrcene, a potential impact compound with herbal and rose notes (Table 4), is biosynthesized via geranyl diphosphate, which undergoes hydrolysis to form geraniol, which is dehydrated and isomerized to produce β-myrcene [61]. The biosynthesis of β-ocimene occurs through the mevalonate and 1-Deoxy-D-Xylulose 5-Phosphate pathways [62]. Another potential impact compound, D-Limonene, identified in three genotypes (Verdim, Alecrim, and CH1), contributes to citrus, orange, lemon, and sweet notes. Sabinene, an impact compound identified only in two genotypes (Verdim and Bicudo), contributes to woody, spicy, citric, terpenic, camphoreous, and pine notes. γ-Terpinene was another impact compound identified only in two genotypes (B01 and Bicudo) and imparts citric, lemon, and herbal notes. Nerol oxide, isolated initially from neroli oil, was identified in four genotypes (Verdim, B01, Bicudo, and Alecrim). This monoterpene is found in many essential oils and contributes to floral, narcissus (*Narcissus* L.), and orange blossom (*Citrus aurantium* L.) notes (Table 4).

Esters accounted for 2.2–4.6% of the total VOC peak areas. These are crucial volatile compounds in many fruits, and most have a strong fruity and floral odor (Table 4). Methyl salicylate, methyl anthranilate, benzyl acetate, and geranyl acetate were identified in all evaluated genotypes. Previously identified in toasted maté [63], methyl salicylate was reported as an important component for the overall tea aroma formation [64]. It has also been identified in Pu-erh tea [65] and oolong tea [66]. Methyl anthranilate contributes to orange blossom and neroli notes (Table 4). The biosynthesis of methyl anthranilate in plants involves an alcohol acyltransferase that catalyzes the formation of methyl anthranilate from anthraniloyl-coenzyme A (CoA) and methanol [67]. Geranyl acetate, a potential impact compound, contributes floral, rose, and lavender notes. Benzyl acetate, a key volatile compound in jasmine tea, contributes to sweet, floral, and jasmine notes [68]. Before Linnaeus classified the *Coffea arabica* plant as such, it was called *Jasminum arabicum* because the flower’s aroma was often confused with jasmine [69] . Geranyl benzoate, identified in four genotypes (B01, Bicudo, 700, and CH1), contributes sweet, rose, and ylang (*Cananga odorata*) notes, whose essential oil has an intensely sweet floral aroma, similar to jasmine [70].

Alcohols accounted for 5.7–37.3% of the total VOC peak areas. Alcohols, in general, contribute to honey, floral, fresh, rose, citrus, and alcohol notes (Table 4). (Z)-3-hexen-1-ol, phenylethyl alcohol, 3-octenol, 2-ethyl-1-hexanol, and benzyl alcohol were identified in all evaluated genotypes. These compounds have been reported as important for black tea [28,47,50,51,52]. Phenylethyl alcohol, a potential impact compound with spicy, rose, lilac, floral, and fresh notes (Table 4), is synthesized in plants via the phenylpropanoid pathway [71]. Benzyl alcohol has also been reported as a key aroma compound in jasmine tea [69]. 2-Butanol, 3-methyl-, and 4-Methylphenethyl alcohols were only identified in the genotype Alecrim and impart floral, balsam, green, rose, and cider notes (Table 4).

Monoterpene alcohols comprised 23.9–48.6% of the total VOC peak areas. They usually add floral, sweet, citrus, herbal, and alcohol notes (Table 4). Linalool, linalool oxide, α-terpineol, geraniol, and nerol were identified in all evaluated genotypes. Linalool, a potential aroma impact compound reported as being key in black tea [28,51] and jasmine tea [69], contributes to citrus, floral, lavender, and sweet notes. Its production in plants involves the mevalonate pathway [72]. Geraniol, another potential impact compound, imparts fruity, rose, and citric notes, and its production in plants occurs through either the methylerythritol–phosphate or the mevalonate pathway [73]. Nerol contributes to sweet, citrus, neroli, and magnolia (*Magnolia* L.) notes (Table 4).

Acids comprised 3.8–7.2% of the total peak areas in coffee flowers. Acids usually add acid, cheesy, sweat, and sour characteristics to the beverage (Table 4). Acetic acid, isovaleric acid, and geranic acid were identified in all genotypes. Valeric acid was only identified in genotype CH1.

Ketones accounted for 0.3–2.5% of the total peak area of the volatile fraction of samples. These compounds generally emit sweet, fruity, rose, and honey notes (Table 4). Because ketones have relatively low odor thresholds, they are thought to play a key role in Pu-erh tea aroma [64,65], and almost allof them have singular odors [59]. Geranyl acetone was the only ketone identified in all coffee flowers. It contributes leafy, magnolia, rose, and woody notes (Table 4). The mevalonate pathway is involved in geranyl acetone production in plants [74]. With geranium and rose notes, benzophenone was identified in three genotypes (Verdim, Alecrim, and CH1).

Furans accounted for 0.2–1.1% of the total VOC peak areas. Furfural was identified in all genotypes. It imparts bread, almond, sweet, caramel, cocoa, and woody characters (Table 4). Dihydroactinidiolide, an important carotenoid-derived impact compound in black tea [75], was detected in four genotypes (B01, Bicudo, Alecrim, and CH1). It has also been previously identified in Pu-erh tea [65]. Pyrazin accounted for about 0.1% of the total peak area of samples. These compounds are known as heat treatment markers [76]. The Maillard reaction during tea manufacturing can generate them [75] and highlights the importance of the drying process for the overall aroma profile. Although none of the coffee flower samples were roasted/toasted, slow drying at low temperatures led to the formation of typical roast aroma compounds, such as methylpyrazine, with nutty, popcorn, brown, roasted, and chocolate notes. This compound was identified in two genotypes (Bicudo and CH1) (Table 4).

Dimethyl sulfide, an organosulfur compound, was detected in all genotypes (accounting for 0.06–0.11% of the total peak area of the volatile fraction). It imparts sulfurous, fishy, seafood, berry, fruity, and vegetable notes (Table 4).

#### 3.4.2. VOC in Coffee Flower Infusions

Considering all infusions, 38 volatile organic compounds (corresponding to 93–96% of the total peak areas of the chromatograms) were accurately identified (Table 4). They were grouped into 7 chemical classes: 12 monoterpenes, 8 aldehydes, 7 monoterpene alcohols, 5 alcohols, 3 esters, 2 ketones, and 1 acid.

Volatile compounds of the monoterpene and monoterpene alcohol classes were most significantly identified in the infusions: all monoterpene alcohol compounds identified in *C. canephora* flowers were identified in their infusions, and 80% of monoterpenes identified in the flowers were identified in the infusions, probably due to good water solubility during the infusion’s preparation. On the other hand, only 40% of the number of ketones, 39% of alcohols, 35% of aldehydes, 23% of esters, and 20% of acids identified in *C. canephora* flowers were identified in their infusions, probably due to volatilization and degradation during the infusion’s preparation. Furans, pyrazins, and organosulfur compounds were not identified in the infusions.

### 3.5. Sensory Evaluation by the Trained Panel

Table 5 presents the main sensory attributes of fragrance, aroma, flavor, and taste/mouthfeel reported for the individual coffee flower infusions by a trained panel of nine tasters.

In general, floral, jasmine, and orange blossom were the most cited sensory attributes for fragrance. For aroma, jasmine, orange blossom, herbal, woody, and sweet were the most cited. Green coffee, woody, jasmine, and orange blossom were the most cited sensory attributes for flavor.

Regarding the attributes considered to be negative, 3 assessors identified soapy notes in two samples, and 2 assessors identified fishy notes in three samples. The soapy note can be attributed to the aldehydes octanal, decanal, and dodecanal, and the fishy note to tetradecanal (aldehyde) and dimethyl sulfide (organosulfur compound) (Table 4).

Although no evaluators noticed notes of magnolia, whose flowers are also white, two compounds that agree with this attribute were identified in the analysis of VOC in all samples evaluated: nerol and geranyl acetone. Notes of other flowers, such as lavender (linalool and geranyl acetate) and geranium (benzophenone), were also identified in the VOC analysis. This may result either from low concentration and/or the high odor threshold of the compounds associated with these attributes or from the fact that these assessors do not experience these aromas habitually, given that only those who are used to consuming those foods or have them in their olfactory memory can recognize them [22]. Table 6 shows the sensory attributes reported by the trained panel and the corresponding volatile compounds identified in this study for coffee flower infusions.

## 4. Conclusions and Final Considerations

This study characterized the major bioactive and volatile composition of *C. canephora* flowers and their infusions. The flowers showed variable and substantial contents of CGA, caffeine, and trigonelline, which were mostly wellextracted when the flowers were infused in hot water. These compositional data suggest great potential for making value-added products with probable health benefits. The lower caffeine content compared to coffee seeds and *Camellia sinensis* teas offers an optional and pleasant hot beverage for consumers sensitive to the stimulant effect of the alkaloid.

Because the bioactive and volatile compounds are not entirely transferred to the infusions, making flour with this raw material is still a potential alternative to use wisely, provided they are pesticide-free and microbiological contamination-free. Additionally, we recommend that further chemical and toxicological studies be carried out on the flowers.

The aroma and flavor profile of the flowers and infusions observed by the panel could be explained by the several volatile compounds identified in this study. From 85 VOC, considering the six evaluated genotypes, 24 were present in all flower samples and infusions and 15 only in all flowers. Among these 39 compounds, 14 are known as potential impact compounds (benzaldehyde, hexanal, heptanal, octanal, nonanal, citronellal, β-myrcene, geranyl acetate, phenylethyl alcohol, 3-octenol, linalool, α-terpineol, geraniol, and acetic acid). Isobutyraldehyde, benzeneacetaldehyde, β-ocimene, trans-alloocimene, methyl anthranilate, benzyl acetate, 2-ethyl-1-hexanol, benzyl alcohol, nerol, and geranyl acetone are additional compounds listed as important for the aroma of similar flowers and probably helped design the coffee flower aroma.

Although different genotypes of *C. canephora* cv. conilon were grown on the same farm, they presented significant differences in VOC composition, sensory characterization, and flavor intensity. This fact deserves attention and should be investigated in additional genotypes and future crops for genotype selection. The possible relationship between the sensory aspects of the flowers and seeds also deserves further investigation.

We hope this work will contribute to the future development of coffee flower aroma and flavor wheels and to the consolidation of coffee flower tea consumption worldwide to stimulate the use of all noble parts of the coffee plant, increasing farmers’ income and supporting the Sustainable Development Agenda for 2030.

## Figures and Tables

**Table 1:** Water content and major bioactive compounds in methanolic extracts of *C. canephora* flowers *.

Samples (Genotypes)	Water Content (%)	*Chlorogenic Acids* (mg/100 g)								
		3-CQA	4-CQA	5-CQA	4-FQA	5-FQA	3,4- diCQA	3,5- diCQA	4,5- diCQA	Total CGA
Verdim R	6.1 ± 0.2 ^c^	20.9 ± 0.5 ^f^	28.8 ± 0.2 ^ef^	224.9 ± 2.1 ^ef^	2.1 ± 0.0 ^e^	20.9 ± 0.2 ^e,f^	7.9 ± 0.0 ^ef^	25.0 ± 0.1 ^f^	12.3 ± 0.0 ^ef^	342.8 ± 1.1 ^f^
B01	7.3 ± 0.3 ^b^	53.9 ± 0.7 ^b^	74.3 ± 0.4 ^b^	580.1 ± 4.0 ^b^	5.3 ± 0.0 ^b^	53.9 ± 0.3 ^b^	20.3 ± 0.1 ^ab^	64.6 ± 0.3 ^b^	31.8 ± 0.1 ^b^	884.3 ± 2.3 ^b^
Bicudo	6.9 ± 0.5 ^b^	45.3 ± 0.3 ^c^	62.4 ± 0.4 ^c^	487.3 ± 2.9 ^c^	4.5 ± 0.0 ^c^	45.3 ± 0.2 ^c^	17.1 ± 0.1 ^c^	54.2 ± 0.2 ^c^	26.7 ± 0.2 ^c^	742.8 ± 1.8 ^c^
Alecrim	7.6 ± 0.5 ^a^	65.8 ± 0.6 ^a^	90.7 ± 0.2 ^a^	708.1 ± 5.3 ^a^	6.5 ± 0.0 ^a^	65.8 ± 0.3 ^a^	24.8 ± 0.2 ^a^	78.8 ± 0.4 ^a^	38.9 ± 0.2 ^a^	1079.4 ± 3.6 ^a^
700	7.0 ± 0.4 ^b^	25.2 ± 0.4 ^e^	34.7 ± 0.3 ^e^	270.7 ± 1.8 ^e^	2.5 ± 0.0 ^e^	25.2 ± 0.1 ^e^	9.5 ± 0.0 ^e^	30.1 ± 0.2 ^e^	14.9 ± 0.0 ^e^	412.6 ± 1.7 ^e^
CH1	6.2 ± 0.1 ^c^	38.8 ± 0.1 ^d^	53.5 ± 0.4 ^d^	417.6 ±2.7 ^d^	3.8 ± 0.0 ^d^	38.8 ± 0.2 ^d^	14.6 ± 0.0 ^cd^	46.5 ± 0.2 ^d^	22.9 ± 0.1 ^cd^	636.6 ± 2.1 ^d^
**Samples** **(Genotypes)**	***Alkaloids* (mg/100 g)**			***Phenolic Acids* (mg/100 g)**						
	**Caffeine**	**Trigonelline**		**Caffeic**	**Ferulic**	***p*-cumaric**	**Vanillic**	**Gallic**	**3,4-** **dihydroxy** **benzoic**	**Total** **phenolic acids**
Verdim R	589.5 ± 2.4 ^bc^	645.2 ± 1.6 ^a^		6.2 ± 0.0 ^e^	4.1 ± 0.0 ^cd^	3.7 ± 0.0 ^e^	6.7 ± 0.0 ^bc^	3.1 ± 0.0 ^cd^	3.3 ± 0.0 ^e^	27.1 ± 0.1 ^d^
B01	745.9 ± 3.1 ^a^	539.3 ± 3.3 ^c^		7.3 ± 0.0 ^d^	5.2 ± 0.0 ^b^	3.9 ± 0.0 ^de^	5.9 ± 0.0 ^d^	2.7 ± 0.0 ^e^	3.7 ± 0.0 ^d^	28.7 ± 0.1 ^d^
Bicudo	543.2 ± 4.0 ^c^	453.8 ± 3.5 ^e^		8.4 ± 0.0 ^b^	6.0 ± 0.0 ^a^	4.1 ± 0.0 ^d^	6.3 ± 0.0 ^c^	3.4 ± 0.0 ^c^	4.3 ± 0.0 ^c^	32.5 ± 0.1 ^c^
Alecrim	621.0 ± 3.6 ^b^	612.7 ± 3.7 ^ab^		9.0 ± 0.0 ^a^	5.8 ± 0.0 ^a^	4.7 ± 0.0 ^c^	7.4 ± 0.0 ^a^	2.9 ± 0.0 ^e^	3.9 ± 0.0 ^d^	33.7 ± 0.1 ^c^
700	515.6 ± 2.4 ^d^	521.5 ± 3.2 ^c^		7.5 ± 0.0 ^d^	5.7 ± 0.0 ^a^	6.2 ± 0.0 ^a^	7.1 ± 0.0 ^ab^	7.9 ± 0.0 ^a^	6.6 ± 0.0 ^a^	41.0 ± 0.2 ^a^
CH1	555.4 ± 3.2 ^c^	502.3 ± 2.2 ^d^		7.9 ± 0.0 ^c^	4.4 ± 0.0 ^c^	5.9 ± 0.0 ^ab^	6.9 ± 0.0 ^b^	7.3 ± 0.0 ^b^	5.2 ± 0.0 ^b^	37.6 ± 0.2 ^ab^

Notes: * values are mean of triplicate analyses ± SD; 3-CQA (3-caffeoylquinic acid); 4-CQA (4-caffeoylquinic acid); 5-CQA (5-caffeoylquinic acid); 4-FQA (4-feruloylquinic acid); 5-FQA (5-feruloylquinic acid); 3,4-diCQA (3,4-dicaffeoylquinic acid); 3,5-diCQA (3,5-dicaffeoylquinic acid); 4,5-diCQA (4,5-dicaffeoylquinic acid). Total chlorogenic acids (CGA): sum of 3-CQA, 4-CQA, 5-CQA, 4-FQA, 5-FQA, 3,4-diCQA, 3,5-diCQA, and 4,5-diCQA. The limit of quantification (LOQ) (peak area equivalent to three times the area of baseline noise) for phenolic acids was 2–3 µg/100 g. Different letters over the bars indicate statistical differences among samples using ANOVA (*p* ≤ 0.05).

**Table 2:** Visual and instrumental color, soluble solids, pH, and titratable acidity of *C. canephora* flowers infusions *.

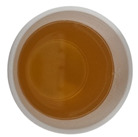 Typical appearance of coffee flower infusions	**Samples** **(Genotypes)**	**Instrumental Color**			**Soluble** **Solids** **(*°Brix*)**	**pH**	**Titratable Acidity** ***(mEq NaOH/L)***
		L*	a*	b*			
	Verdim R	87.25 ± 0.19 ^b^	−4.19 ± 0.06 ^c^	8.40 ± 0.15 ^ab^	0.2 ± 0.0 ^a^	5.6 ± 0.0 ^a^	0.08 ± 0.004 ^b^
	B01	85.90 ± 0.16 ^d^	−4.32 ± 0.08 ^a^	8.34 ± 0.12 ^b^	0.2 ± 0.0 ^a^	5.4 ± 0.0 ^b^	0.10 ± 0.003 ^a^
	Bicudo	88.25 ± 0.21 ^ab^	−4.25 ± 0.05 ^b^	8.22 ± 0.13 ^c^	0.2 ± 0.0 ^a^	5.5 ± 0.0 ^a^	0.08 ± 0.003 ^b^
	Alecrim	87.66 ± 0.13 ^b^	−4.18 ± 0.04 ^c^	8.41 ± 0.14 ^a^	0.2 ± 0.0 ^a^	5.3 ± 0.0 ^b^	0.10 ± 0.004 ^a^
	700	86.33 ± 0.15 ^c^	−4.28 ± 0.05 ^ab^	8.38 ± 0.11 ^ab^	0.2 ± 0.0 ^a^	5.6 ± 0.0 ^a^	0.08 ± 0.002 ^b^
	CH1	89.44 ± 0.12 ^a^	−4.31 ± 0.06 ^a^	8.45 ± 0.11 ^a^	0.2 ± 0.0 ^a^	5.5 ± 0.0 ^a^	0.08 ± 0.004 ^b^

Notes: * values are mean of triplicate readings ± SD. Different letters over the bars indicate statistical differences among samples by ANOVA (*p* ≤ 0.05). L* = lightness measured as brightness, with 100 and 0 values corresponding to absolute white and black, respectively; a* and b* = chromaticity (+a* red and −a* green, +b* yellow and -b* blue) [43].

**Table 3:** Major bioactive compounds in *C. canephora* flower infusions *.

Samples (Genotypes)	Caffeine	Trigonelline	Total CQA	Total FQA	Total diCQA	Total CGA
	*mg/100 mL*					
Verdim	5.1 ± 0.02 ^bc^	4.8 ± 0.02 ^b^	2.28 ± 0.01 ^de^	0.38 ± 0.00 ^e^	0.19 ± 0.00 ^de^	2.8 ± 0.01 ^e^
B01	7.1 ± 0.03 ^a^	3.9 ± 0.01 ^c^	5.60 ± 0.02 ^b^	0.92 ± 0.01 ^b^	0.47 ± 0.00 ^b^	7.0 ± 0.02 ^b^
Bicudo	4.9 ± 0.02 ^c^	3.4 ± 0.01 ^d^	4.82 ± 0.02 ^c^	0.79 ± 0.00 ^c^	0.40 ± 0.00 ^b^	6.0 ± 0.02 ^bc^
Alecrim	5.5 ± 0.04 ^b^	4.6 ± 0.02 ^a^	7.35 ± 0.03 ^a^	1.21 ± 0.01 ^a^	0.61 ± 0.00 ^a^	9.2 ± 0.03 ^a^
700	4.7 ± 0.03 ^c^	4.0 ± 0.01 ^c^	2.88 ± 0.02 ^d^	0.47 ± 0.00 ^d^	0.24 ± 0.00 ^d^	3.4 ± 0.02 ^d^
CH1	5.2 ± 0.02 ^b^	3.5 ± 0.01 ^d^	4.39 ± 0.02 ^c^	0.72 ± 0.00 ^c^	0.37 ± 0.00 ^c^	5.5 ± 0.01 ^c^

Notes: * values are mean of duplicate analyses ± SD. Total CQA: sum of 3-CQA (3-caffeoylquinic acid) + 4-CQA (4-caffeoylquinic acid) + 5-CQA (5-caffeoylquinic acid). Total FQA: sum of 4-FQA (4-feruloylquinic acid) + 5-FQA (5-feruloylquinic acid). Total diCQA: sum of 3,4-diCQA (3,4-dicaffeoylquinic acid) + 3,5-diCQA (3,5-dicaffeoylquinic acid) + 4,5-diCQA (4,5-dicaffeoylquinic acid). Total CGA: sum of total CQA + total FQA + total diCQA. Different letters over the bars indicate statistical differences among samples by ANOVA (*p* ≤ 0.05).

**Table 4:** Volatile organic compounds identified in coffee flowers and their infusions, as well as their classical odor description.

Volatile Compounds	Odor Description [45,46]	#CAS	LRI	ELRI	Verdim		B01		Bicudo		Alecrim		700		CH1	
					FLO	INF	FLO	INF	FLO	INF	FLO	INF	FLO	INF	FLO	INF
**Aldehydes**																
Acetaldehyde	Pungent, ether, fresh, fruity, musty	75-07-0	962	962	■ ^a^	□	■ ^a^	□	■ ^a^	□	■ ^a^	□	■ ^a^	□	■ ^a^	□
Isobutyraldehyde	Pungent, malt, green, fresh, aldehydic, floral	78-84-2	913	913	■ ^a^	□	■ ^a^	□	■ ^a^	□	■ ^a^	□	■ ^a^	□	■ ^a^	□
Isovaleraldehyde	Ethereal, aldehydic, chocolate, peach, fatty	590-86-3	925	925	■ ^a^	□	■ ^a^	□	■ ^a^	□	■ ^a^	□	■ ^a^	□	■ ^a^	□
α-Methylbutanal	Cocoa, coffee, nutty, malty, fermented, fatty, alcoholic	96-17-3	922	922	■ ^a^	■ ^a^	■ ^a^	■ ^a^	■ ^a^	■ ^a^	■ ^a^	■ ^a^	■ ^a^	■ ^a^	■ ^a^	■ ^a^
3-Methyl-2-butenal	Sweet, fruity, pungent, brown, nutty, almond, cherry	107-86-8	911	923	□	□	■ ^a^	□	□	□	□	□	□	□	□	□
Benzaldehyde *	Almond, burnt sugar, fruity, cherry, sweet	100-52-7	948	948	■ ^a^	■ ^a^	■ ^a^	■ ^a^	■ ^a^	■ ^a^	■ ^a^	■ ^a^	■ ^a^	■ ^a^	■ ^a^	■ ^a^
Benzeneacetaldehyde	Honey, floral, sweet, fermented, chocolate, earthy, green	122-78-1	929	931	■ ^a^	■ ^a^	■ ^a^	■ ^a^	■ ^a^	■ ^a^	■ ^a^	■ ^a^	■ ^a^	■ ^a^	■ ^a^	■ ^a^
Pentanal	Fermented, bready, fruity, berry, nutty, malt, pungent	110-62-3	810	810	□	□	□	□	□	□	■ ^ac^	■ ^ac^	□	□	□	□
Hexanal *	Grass, fresh, leafy, aldehydic, fruity, fatty, tallow	66-25-1	948	948	■ ^ac^	□	■ ^ac^	□	■ ^ac^	□	■ ^ac^	□	■ ^ac^	□	■ ^ac^	□
2-Hexenal, (E)-	Sharp, fresh, leafy, herbal, spicy	6728-26-3	947	952	■ ^b^	□	■ ^b^	□	■ ^b^	□	■ ^a^	□	■ ^b^	□	■ ^a^	□
Heptanal *	Fatty, rancid, citric, fresh, aldehydic, herbal, wine-lee	111-71-7	797	797	■ ^a^	□	■ ^a^	□	■ ^a^	□	■ ^a^	□	■ ^a^	□	■ ^a^	□
Octanal *	Citric, lemon, orange, herbal, fresh, aldehydic, soapy	124-13-0	855	891	■ ^a^	□	■ ^a^	□	■ ^a^	□	■ ^a^	□	■ ^a^	□	■ ^a^	□
Nonanal *	Citric, fresh, orange, green, rose, aldehydic, fatty	124-19-6	782	784	■ ^ac^	■ ^ac^	■ ^ac^	■ ^ac^	■ ^ac^	■ ^ac^	■ ^ac^	■ ^ac^	■ ^ac^	■ ^ac^	■ ^ac^	■ ^ac^
Decanal *	Sweet, citric, floral, soapy, orange peel, aldehydic	112-31-2	912	912	■ ^a^	□	□	□	□	□	■ ^a^	□	■ ^a^	□	■ ^a^	□
Dodecanal *	Soapy, waxy, aldehydic, citric, orange, green, floral	112-54-9	754	766	□	□	□	□	□	□	□	□	■ ^b^	□	□	□
Tetradecanal	Fatty, waxy, dairy, creamy, fishy, fruity, pear, citric	124-25-4	911	911	□	□	□	□	□	□	■ ^b^	□	■ ^b^	□	□	□
β-Citral (neral)	Sweet, citric, lemon, lemon peel	106-26-3	938	938	■ ^a^	■ ^a^	■ ^a^	■ ^a^	■ ^a^	■ ^a^	■ ^a^	■ ^a^	■ ^a^	■ ^a^	■ ^a^	■ ^a^
α-Citral (geranial)	Citric, lemon	141-27-5	931	932	■ ^b^	■ ^a^	■ ^a^	■ ^a^	■ ^a^	■ ^a^	■ ^a^	■ ^a^	■ ^a^	■ ^a^	■	■ ^a^
Citral	Fresh, lemon peel, sweet, tart, green	5392-40-5	879	885	□	□	□	□	□	□	■ ^b^	□	□	□	□	□
Citronellal *	Sweet, floral, rose, herbal, waxy, aldehydic, citric	106-23-0	606	606	■ ^a^	■ ^a^	■ ^a^	■ ^a^	■ ^a^	■ ^a^	■ ^a^	■ ^a^	■ ^a^	■ ^a^	■ ^a^	■ ^a^
Lilac aldehyde A	Floral, fresh	53447-46-4	805	817	□	□	□	□	□	□	□	□	■ ^b^	□	□	□
Lilac aldehyde C	Sweet, floral	53447-48-6	791	796	□	□	□	□	□	□	□	□	■ ^b^	□	□	□
Lilac aldehyde D	Sweet, floral	53447-47-5	636	672	□	□	□	□	□	□	□	□	■ ^b^	□	■ ^b^	□
**Monoterpenes**																
*D*-Limonene *	Citrus, orange, lemon, fresh, sweet	5989-27-5	927	927	■ ^a^	■ ^a^	□	□	□	□	■ ^a^	■ ^a^	□	□	■ ^a^	■ ^a^
β-Myrcene *	Balsamic, musty, herbal, woody, spicy, rose, carrot	123-35-3	961	962	■ ^ac^	■ ^ac^	■ ^ac^	■ ^ac^	■ ^ac^	■ ^ac^	■ ^ac^	■ ^ac^	■ ^ac^	■ ^ac^	■ ^ac^	■ ^ac^
α-Phellandrene	Citric, herbal, terpenic, woody, black pepper, spicy, minty	99-83-2	855	868	■ ^a^	■ ^a^	■ ^a^	■ ^a^	■ ^a^	■ ^a^	■ ^a^	■ ^a^	■ ^a^	■ ^a^	■ ^a^	■ ^a^
β-Phellandrene	Minty, terpenic	555-10-2	842	842	□	□	□	□	□	□	□	□	□	□	■ ^b^	■ ^b^
*p*-Cymene	Fresh, citric, woody, terpenic, lemon, spicy, cumin, cilantro	99-87-6	896	896	■ ^b^	■ ^b^	■ ^b^	■ ^b^	■ ^b^	■ ^b^	□	□	■ ^b^	■ ^b^	□	□
β*-*Cymene	Fresh, citric, terpenic, woody, spicy	535-77-3	886	886	□	□	□	□	■ ^b^	□	■ ^b^	□	□	□	■ ^b^	□
Sabinene *	Woody, spicy, citric, terpenic, green, camphoreous, pine	3387-41-5	819	822	■ ^b^	■ ^b^	□	□	■ ^b^	■ ^b^	□	□	□	□	□	□
β-Ocimene	Citric, tropical, herbal, terpenic, woody, sweet	13877-91-3	918	922	■ ^b^	■ ^b^	■ ^b^	■ ^b^	■ ^b^	■ ^b^	■ ^b^	■ ^b^	■ ^b^	■ ^b^	■ ^b^	■ ^b^
*trans*-Alloocimene	Sweet, floral, nutty, peppery, herbal, tropical	673-84-7	811	811	■ ^b^	■ ^b^	■ ^b^	■ ^b^	■ ^b^	■ ^b^	■ ^b^	■ ^b^	■ ^b^	■ ^b^	■ ^b^	■ ^b^
Nerol oxide	Green, narcissus, celery, floral, orange blossom, minty	1786-08-9	698	698	■ ^a^	■ ^a^	■ ^a^	■ ^a^	■ ^a^	■ ^a^	■ ^a^	■ ^a^	□	□	□	□
α-Terpinene	Citric, woody, terpenic, camphoreous, spicy, medicinal	99-86-5	840	849	■ ^b^	■ ^b^	■ ^b^	■ ^b^	■ ^b^	■ ^a^	■ ^b^	■ ^b^	■ ^b^	■ ^b^	■ ^b^	■ ^b^
γ-Terpinene *	Oily, woody, citric, lemon, tropical, herbal, gasoline	99-85-4	860	871	□	□	■ ^b^	■ ^b^	■ ^b^	■ ^b^	□	□	□	□	□	□
α-Thujene	Woody, green, herbal	2867-05-2	911	911	□	□	□	□	■ ^a^	■ ^b^	■ ^a^	■ ^b^	■ ^a^	■ ^a^	■ ^a^	■ ^a^
α-Terpinolene *	Sweet, fresh, pine, citric, woody, lemon peel	586-62-9	964	964	□	□	□	□	■ ^b^	□	□	□	□	□	□	□
Isoterpinolene	Herbal, woody	586-63-0	795	795	□	□	□	□	□	□	□	□	■ ^b^	■ ^b^	■ ^b^	■ ^b^
**Esters**																
Methyl salicylate	Sweet, wintergreen, phenolic, camphoreous, peppermint	119-36-8	899	901	■ ^a^	■ ^a^	■ ^a^	■ ^a^	■ ^a^	■ ^a^	■ ^a^	■ ^a^	■ ^a^	■ ^a^	■ ^a^	■ ^a^
Methyl anthranilate	Fruity, grape, orange blossom, neroli	134-20-3	760	760	■ ^a^	■ ^a^	■ ^a^	■ ^a^	■ ^a^	■ ^a^	■ ^a^	■ ^a^	■ ^a^	■ ^a^	■ ^a^	■ ^a^
Methyl myristate	Fatty, waxy, petal	124-10-7	836	834	□	□	□	□	□	□	□	□	■ ^a^	□	□	□
Methyl palmitate	Oily, waxy, fatty, orris	112-39-0	762	772	□	□	□	□	□	□	■ ^a^	□	■ ^a^	□	■ ^a^	□
Methyl phenyl acetate	Sweet, floral, honey, spicy, waxy, almond	101-41-7	662	662	■ ^a^	□	□	□	□	□	□	□	■ ^a^	□	□	□
Benzyl acetate	Sweet, floral, fruity, jasmine, boiled vegetable	140-11-4	904	904	■ ^a^	□	■ ^a^	□	■ ^a^	□	■ ^a^	□	■ ^a^	□	■ ^a^	□
Benzyl salicylate	Balsam, clean, herbal, oily, sweet	118-58-1	860	901	□	□	□	□	□	□	□	□	□	□	■ ^a^	■ ^a^
Butyl benzoate	Mild, amber, balsam, fruity	136-60-7	814	817	■ ^b^	□	■ ^b^	□	■ ^b^	□	□	□	■ ^a^	□	■ ^b^	□
Isobutyl benzoate	Sweet, fruity, musty, powdery, balsam	120-50-3	841	902	□	□	□	□	□	□	■ ^b^	□	□	□	□	□
Geranyl acetate*	Floral, rose, lavender, green, waxy	105-87-3	842	842	■ ^b^	□	■ ^b^	□	■ ^b^	□	■ ^b^	□	■ ^b^	□	■ ^b^	□
Geranyl benzoate	Sweet, amber, ylang, rose	94-48-4	873	881	□	□	■ ^b^	□	■ ^a^	□	□	□	■ ^a^	□	■ ^a^	□
Geranyl formate	Fresh, rose, neroli, tea, green	105-86-2	850	850	□	□	□	□	■ ^a^	□	□	□	□	□	□	□
2-Methybutyl propionate	Sweet, fruity, ethereal, rummy	2438-20-2	758	758	□	□	■ ^b^	□	□	□	■ ^b^	□	■ ^b^	□	■ ^b^	□
**Alcohol**																
Ethanol	Alcoholic, ethereal, medicinal, sweet	64-17-5	955	955	■ ^a^	□	■ ^a^	□	■ ^a^	□	■ ^a^	□	□	□	□	□
2-Methyl-1-butanol	Ethereal, alcoholic, fatty, greasy, winey, whiskey, cocoa	137-32-6	693	752	■ ^b^	□	■ ^a^	□	■ ^b^	□	■ ^a^	□	□	□	■ ^a^	□
(Z)-3-hexen-1-ol	Grass, fresh, foliage, herbal, oily, melon, pungent	928-96-1	941	942	■ ^b^	□	■ ^b^	□	■ ^b^	□	■ ^b^	□	■ ^b^	□	■ ^b^	□
Phenylethyl alcohol *	Honey, spicy, rose, lilac, floral, sweet, fresh	60-12-8	939	966	■ ^a^	□	■ ^a^	□	■ ^a^	□	■ ^a^	□	■ ^a^	□	■ ^a^	□
n-Tridecan-1-ol	Musty	112-70-9	776	776	■ ^a^	□	■ ^b^	□	□	□	□	□	■ ^b^	□	□	□
3-Octenol *	Mushroom, earthy, green, oily, fungal, raw chicken	20125-85-3	861	867	■ ^a^	■ ^b^	■ ^a^	■ ^b^	■ ^a^	■ ^b^	■ ^a^	■ ^b^	■ ^a^	■ ^b^	■ ^a^	■ ^b^
2-Ethyl-1-hexanol	Rose, green, citric, fresh, floral, oily, sweet	104-76-7	901	908	■ ^b^	■ ^b^	■ ^b^	■ ^b^	■ ^b^	■ ^b^	■ ^b^	■ ^b^	■ ^b^	■ ^b^	■ ^b^	■ ^b^
Benzyl alcohol	Floral, rose, phenolic, balsam, sweet, fruity	100-51-6	922	925	■ ^a^	■ ^b^	■ ^a^	■ ^b^	■ ^a^	■ ^b^	■ ^a^	■ ^a^	■ ^b^	■ ^a^	■ ^b^	■ ^a^
2-Butanol, 3-methyl-	Musty, alcoholic, fusel, vegetable, cider, cocoa, cheesy	598-75-4	773	821	□	□	□	□	□	□	■ ^b^	■ ^b^	□	□	□	□
4-Methylphenethyl alcohol	Floral, balsam, rose, green	699-02-5	866	866	□	□	□	□	□	□	■ ^a^	■ ^a^	□	□	□	□
1-Hexanol	Ethereal, fusel, oily, fruity, alcoholic, sweet, green	111-27-3	917	917	□	□	■ ^a^	□	■ ^a^	□	■ ^b^	□	■ ^a^	□	□	□
2-Heptanol	Fresh, lemon balm, herbal, sweet, floral, fruity	543-49-7	913	920	□	□	□	□	■ ^b^	□	■ ^a^	□	□	□	■ ^a^	□
2-Nonen-1-ol	Sweet, fatty, melon, cucumber, vegetable	22104-79-6	710	710	□	□	□	□	□	□	■ ^b^	□	□	□	□	□
**Monoterpenes alcohol**																
Linalool *	Citrus, floral, blueberry, lavender, bois de rose, sweet	78-70-6	946	946	■ ^ac^	■ ^ac^	■ ^ac^	■ ^ac^	■ ^ac^	■ ^ac^	■ ^ac^	■ ^ac^	■ ^ac^	■ ^ac^	■ ^ac^	■ ^ac^
Linalool oxide	Floral, woody, musty, fenchyl, herbal, alcohol	60047-17-8	917	917	■ ^b^	■ ^b^	■ ^b^	■ ^b^	■ ^b^	■ ^b^	■ ^b^	■ ^b^	■ ^b^	■ ^b^	■ ^b^	■ ^b^
*cis*-Linalool oxide	Earthy, floral, sweet, woody	5989-33-3	669	669	■ ^a^	■ ^b^	□	□	■ ^a^	■ ^b^	■ ^a^	■ ^b^	■ ^a^	■ ^b^	■ ^a^	■ ^b^
*trans*-Linalool oxide	Floral	34995-77-2	840	926	■ ^b^	■ ^b^	■ ^b^	■ ^b^	■ ^b^	■ ^b^	□	□	■ ^b^	■ ^b^	■ ^b^	■ ^b^
α-Terpineol *	Oil, anise, mint, lemon, citric	98-55-5	853	875	■ ^bc^	■ ^bc^	■ ^bc^	■ ^bc^	■ ^bc^	■ ^bc^	■ ^bc^	■ ^bc^	■ ^bc^	■ ^bc^	■ ^bc^	■ ^bc^
Geraniol *	Sweet, floral, fruity, rose, waxy, citric	106-24-1	955	956	■ ^a^	■ ^a^	■ ^a^	■ ^a^	■ ^b^	■ ^a^	■ ^a^	■ ^a^	■ ^a^	■ ^b^	■ ^a^	■ ^a^
Nerol	Sweet, neroli, citric, magnolia	106-25-2	943	945	■ ^a^	■ ^a^	■ ^a^	■ ^a^	■ ^a^	■ ^a^	■ ^a^	■ ^a^	■ ^a^	■ ^a^	■ ^a^	■ ^a^
**Acids**																
Acetic acid *	Acidic, sour, pungent, vinegar	64-19-7	922	937	■ ^a^	□	■ ^a^	□	■ ^a^	□	■ ^a^	□	■ ^a^	□	■ ^a^	□
Isovaleric acid	Sweat, acidic, rancid, stinky, feet, cheesy, fruity	503-74-2	885	885	■ ^a^	□	■ ^a^	□	■ ^a^	□	■ ^a^	□	■ ^a^	□	■ ^a^	□
Valeric acid	Acidic, sharp, cheesy, sour, milky, tobacco, fruity	109-52-4	844	858	□	□	□	□	□	□	□	□	□	□	■ ^a^	□
Caproic acid	Sweat, sour, fatty, cheesy	142-62-1	875	887	■ ^a^	□	■ ^a^	□	■ ^a^	□	■ ^a^	□	■ ^b^	□	□	□
Geranic acid	Dry, weedy, acidic, green, moldy, feet, woody	4613-38-1	892	894	■ ^a^	■ ^a^	■ ^a^	■ ^a^	■ ^b^	■ ^b^	■ ^a^	■ ^a^	■ ^a^	■ ^a^	■ ^a^	■ ^a^
**Ketones**																
Geranyl acetone	Magnolia, rose, leafy, fresh, fruity, woody, tropical	689-67-8	737	765	■ ^b^	■ ^b^	■ ^b^	■ ^b^	■ ^a^	■ ^a^	■ ^b^	■ ^a^	■ ^b^	■ ^a^	■ ^b^	■ ^a^
Benzophenone	Balsam, herbal, rose, metallic, geranium	119-61-9	745	747	■ ^b^	■ ^a^	□	□	□	□	■ ^b^	■ ^a^	□	□	■ ^b^	■ ^a^
Lavender lactone	Fruity, minty	1073-11-6	889	889	□	□	■ ^a^	□	□	□	□	□	□	□	□	□
Acetyl valeryl	Buttery, cheesy, oily	96-04-8	792	792	□	□	□	□	■ ^a^	□	□	□	□	□	□	□
**Furans**																
Furfural	Bread, almond, sweet, brown, woody, caramellic	98-01-1	926	937	■ ^a^	□	■ ^a^	□	■ ^a^	□	■ ^a^	□	■ ^a^	□	■ ^a^	□
Dihydroactinidioide *	Musk, coumarin	17092-92-1	854	869	□	□	■ ^a^	□	■ ^a^	□	■ ^a^	□	■ ^a^	□	□	□
Sedanolide	Herbal, celery	6415-59-4	636	636	□	□	□	□	■ ^a^	□	□	□	□	□	□	□
**Organosulfur**																
Dimethyl sulfide	Sulfurous, onion, sweet, cabbage, tomato, green, radish, creamy, fishy, seafood, berry, fruity, vegetable	75-18-3	943	951	■ ^a^	□	■ ^a^	□	■ ^a^	□	■ ^a^	□	■ ^a^	□	■ ^a^	□
**Pyrazin**																
Methylpyrazine	Nutty, popcorn, brown, musty, earthy, roasted, chocolate	109-08-0	891	891	□	□	□	□	■ ^b^	□	□	□	□	□	■ ^a^	□

**Note:** FLO: flower; INF: infusion. Odor description according to Flavornet [45] and The Good Scents Company Information System [46]. * Impact compounds according to Wang et al. [28]; Schieberle et al. [47]; Araújo et al. [48]; Márquez et al. [49]; Magagna et al. [50]; Kang et al. [51]; Yang et al. [52]; Steger et al. [53]; Mei et al. [54]. CAS# (Chemical Abstracts Service) Registry Number, available in the NIST database [55]; ELRI: Experimental Linear Retention Index; LRI: Linear Retention Index based on the literature and NIST database [55]. ^a^ Compounds identified with probability more than 50%.^b^ Compounds that provided a match factor higher than 600 and a match factor versus reversed match factor ratio greater than 0.8. ^c^ Compounds identified by comparison with standards; ■ compound identified in the sample; □ compound not identified in the sample.

**Table 5:** The main sensory attributes of fragrance, aroma, flavor, and taste/mouthfeel reported for the individual coffee flower infusions by the sensory panel.

Samples (Genotypes)	*Fragrance*	*Aroma*	*Flavor*	*Taste/Mouthfeel*
**Verdim**	Citric, floral, jasmine	Floral, fermented, soap, jasmine, sweet, fresh, metallic	Green coffee, woody, black tea, toasted leaf, floral, cooked vegetable	Sweet, astringent
**B01**	Citric, floral, jasmine, orange blossom	Honey, sweet, soap, black tea, jasmine	Jasmine, orange blossom, herbal, green coffee, honey, toasted leaf	Sweet
**Bicudo**	Floral, jasmine, orange blossom	Woody, herbal, green tea, lemon balm, anise, floral, orange blossom, sweet, fresh	Herbal, green coffee, woody, black tea, fruity, red fruits, honey, peach	Sweet
**Alecrim**	Floral, herbal, jasmine, orange blossom	Herbal, musty, woody, toasted leaf, metallic, medicinal, fishy	Herbal, green coffee, honey, red fruits, black currant/blackberry	Sweet
**700**	Citric, lemon balm, floral, jasmine, orange blossom	Herbal, jasmine, anise, lemon balm, medicinal, fishy	Herbal, toasted leaf, woody, caramelized	Sweet, astringent
**CH1**	Floral, jasmine, orange blossom	Woody, herbal, fermented, musty fishy, sweet	Herbal, green coffee, jasmine, orange blossom, woody, sweet	Sweet

**Table 6:** Attributes perceived by the trained panel and the corresponding volatile compounds identified in this study.

Fragrance, Aroma, and Flavor Attributes	Corresponding Volatile Organic Compounds	References
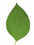 Herbal	Isobutyraldehyde; Benzeneacetaldehyde; Heptanal *; Octanal *; Nonanal *; Dodecanal; Citral; α-Phellandrene; Sabinene; β-Ocimene; *trans*-Alloocimene; Nerol oxide; γ-Terpinene; α-Thujene; Isoterpinolene; Methyl salicylate; Benzyl acetate; Benzyl salicylate; Geranyl formate; (Z)-3-hexen-1-ol; 2-Ethyl-1-hexanol; 2-Butanol, 3-methyl-; 4-Methylphenethyl alcohol; 1-Hexanol; 2-Heptanol; 2-Nonen-1-ol; Linalool *; Linalool oxide; Geranic acid; Benzophenone	[45,46,51,53,54,75,76,77]
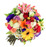 Floral	Isobutyraldehyde; Benzeneacetaldehyde; Lilac aldehyde A; Lilac aldehyde C; Lilac aldehyde D; Decanal *; Dodecanal; Citronellal; *trans*-Alloocimene; Nerol oxide; Methyl phenyl acetate; Benzyl acetate; Geranyl acetate; Phenylethyl alcohol; Benzyl alcohol; 4-Methylphenethyl alcohol; 2-Heptanol; Linalool *; Linalool oxide; *cis*-Linalool oxide; *trans*-Linalool oxide; Geraniol	[45,46,53,54,76,78,79]
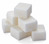 Sweet	3-Methyl-2-butenal; Benzeneacetaldehyde; Decanal *; Citral; Citronellal; Lilac aldehyde C; Lilac aldehyde D; *trans*-Alloocimene; D-Limonene *; α-Terpinolene; Methyl salicylate; Methyl phenyl acetate; Benzyl acetate; Isobutyl benzoate; Geranyl benzoate; 2-Methybutyl propionate; Ethanol; Phenylethyl alcohol; 2-Ethyl-1-hexanol; Benzyl alcohol; 1-Hexanol; 2-Heptanol; 2-Nonen-1-ol; Linalool *;*cis*-Linalool oxide; Geraniol; Nerol; Furfural; Dimethyl sulfide	[45,46,53,76,78]
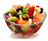 Fruity	Acetaldehyde; 3-Methyl-2-butenal; Benzaldehyde *; Pentanal; Hexanal *; Tetradecanal; Methyl anthranilate; Benzyl acetate; Butyl benzoate; Isobutyl benzoate; 2-Methybutyl propionate; Benzyl alcohol; 1-Hexanol; 2-Heptanol; Geraniol; Isovaleric acid; Valeric acid; Geranyl acetone; Lavender lactone; Dimethyl sulfide	[45,46,53,66,76,78]
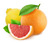 Citric	Heptanal *; Octanal *; Nonanal *; Decanal *; Dodecanal; Tetradecanal; β-Citral (neral); α-Citral (geranial); Citronellal; α-Phellandrene; p-Cymene; β-Cymene; Sabinene; β-Ocimene; α-Terpinene; γ-Terpinene; D-Limonene *; α-Terpinolene; 2-Ethyl-1-hexanol; Linalool *; α-Terpineol *; Geraniol; Nerol	[45,46,52,53]
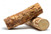 Woody	β-Myrcene *; α-Phellandrene; *p*-Cymene; β-Cymene; Sabinene; β-Ocimene; α-Terpinene; γ-Terpinene; α-Thujene; α-Terpinolene; Isoterpinolene; Linalool oxide;*cis*-Linalool oxide; Geranic acid; Geranyl acetone; Furfural	[45,46,52,53,54,76,78]
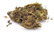 Musty	Acetaldehyde; Isobutyl benzoate; n-Tridecan-1-ol; 2-Butanol, 3-methyl-; Linalool oxide; Methylpyrazine; Geranic acid	[45,46]
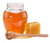 Honey	Benzeneacetaldehyde; Methyl phenyl acetate; Phenylethyl alcohol	[45,46]
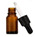 Medicinal	α-Terpinene; Ethanol; Acetaldehyde; Isovaleraldehyde; 2-Methybutyl propionate; 2-Methyl-1-butanol; 1-Hexanol	[52,53]
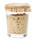 Fermented	α-Methylbutanal; Benzeneacetaldehyde; Pentanal	[45,46,80,81]
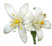 Orange blossom	Methyl anthranilate; Geranyl formate; Nerol	[45,46,56,59,60]
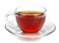 Black tea	Geranyl formate; Benzaldehyde; Hexanal; (Z)-3-hexen-1-ol; Phenylethyl alcohol; 3-Octenol; 2-Ethyl-1-hexanol; Benzyl alcohol	[28,45,46,47,51,52]
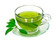 Green tea	Geranyl formate	[45,46,59,75]
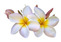 Jasmine	Benzyl acetate; Benzyl alcohol; Linalool	[45,46,69]
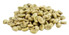 Green coffee	Hexanal; Benzaldehyde; Hexanoic acid	[82]
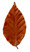 Toasted leaf	Methylpyrazine; Furfural	[45,46,48,49,53,54]
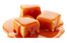 Caramellic	Furfural; Benzaldehyde *	[45,46]
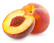 Peach	Isovaleraldehyde	[45,46]
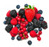 Red fruits	Pentanal; Linalool *; Dimethyl sulfide	[45,46]
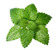 Lemon balm	2-Heptanol	[45,46]
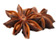 Anise	α-Terpineol *	[45,46]
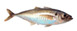 Fishy	Tetradecanal; Dimethyl sulfide	[45,46]
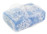 Soapy	Octanal *; Decanal *; Dodecanal	[45,46]

Note: * Potential impact compounds according to the literature.

## Data Availability

Data supporting the reported results are available upon request.
